# Tinnitus is associated with improved cognitive performance and speech perception–Can stochastic resonance explain?

**DOI:** 10.3389/fnagi.2022.1073149

**Published:** 2022-12-16

**Authors:** Achim Schilling, Patrick Krauss

**Affiliations:** ^1^Neuroscience Lab, University Hospital Erlangen, Erlangen, Germany; ^2^Cognitive Computational Neuroscience Group, University of Erlangen-Nurnberg, Erlangen, Germany; ^3^Linguistics Lab, University of Erlangen-Nurnberg, Erlangen, Germany; ^4^Pattern Recognition Lab, University of Erlangen-Nurnberg, Erlangen, Germany

**Keywords:** tinnitus, auditory perception, speech perception, stochastic resonance (SR), cognition, hearing loss, central noise, cognitive decline

Subjective tinnitus is a perceived sound in the absence of any objective sound source. This phantom perception has severe consequences, ranging from insomnia to depression or even suicide. Furthermore, tinnitus is assumed to accelerate cognitive decline. However, a recent study showed that in non-hispanic elderly people, tinnitus is associated with a better cognitive function compared to an age- matched control group.

This finding is counter-intuitive, as tinnitus is highly correlated with hearing loss, and hearing loss is highly correlated with cognitive decline. So how is it possible that a phantom sound causing unwanted and severe side effects is associated with decreased cognitive decline?

We argue that tinnitus is a side effect of a processing mechanism in the auditory system to compensate for reduced auditory input by exploiting a phenomenon called stochastic resonance. In particular, the auditory system uses internally generated neural noise from the somatosensory system to lift a sub-threshold auditory signal above the detection threshold. We could already show in computer simulations that this mechanism has the potential to significantly increase speech perception in hearing impaired people.

We hypothesize that the decreased cognitive decline is a direct consequence of an improved speech perception and less cognitive deprivation due to the stochastic resonance based mechanism of improving hearing ability and speech perception on the one hand and as a side effect causing tinnitus on the other hand.

## Introduction

In our aging society the impairment of cognitive functions named cognitive decline is a big issue that recently gets a lot of attention (see e.g., Burns and Zaudig, 2002; kulak-Bejda et al., 2021). However, cognition is an abstract concept (Morris et al., 1999), which summarizes a lot of different cognitive functions such as processing speed, memory, reasoning, and executive functions such as speech production (Deary et al., 2009). These functions are tested with a bunch of different tasks, such as tests on oral naming of pictures, the recognition of animal silhouettes, or tests on verbal fluency, to test phonological as well as semantic cognitive abilities (Bird et al., 2004; Cullen et al., 2007). It is common knowledge that in elderly people cognitive function are more and more decreased, although it is not obvious which exact factors lead to the cognitive decline. Thus, e.g., dementia leads to cognitive decline, but cognitive decline in turn can also drive dementia worsening (Marioni et al., 2015). Already in 1989, it has been shown that hearing loss can increase the probability of dementia in elderly people (Uhlmann et al., 1989), suggesting that hearing loss plays a crucial role in age related cognitive decline.

## Hearing loss reduces speech perception and cognitive performance

Hearing loss is an important factor driving cognitive decline through several pathways (Uhlmann et al., 1989; Lin et al., 2013; Fortunato et al., 2016; Uchida et al., 2019). Up to now, it is not entirely clear *via* which mechanism hearing loss promotes cognitive decline (Fulton et al., 2015; Jafari et al., 2021). There exist four major theories trying to explain the correlation of hearing loss and cognitive decline: the cognitive-load-on perception-hypothesis, the common-cause-hypothesis, the sensory-deprivation-hypothesis, and the information-degradation-hypothesis (Wayne and Johnsrude, 2015).

The cognitive-load-on-perception hypothesis, which suggests that hearing loss is a result of cognitive decline does not fit to most of the observations (see e.g., Lindenberger and Baltes, 1994).

The common-cause-hypothesis suggests an unknown cause leading coincidentally to cognitive decline as well as hearing loss. However, it is not clear which cause this could be (Wayne and Johnsrude, 2015) and thus, also this theory has low explanatory power.

The sensory-deprivation-theory explains cognitive decline as a direct consequence of sensory deprivation, which means that neuroplastic changes occur, which favor sensory perception at the expense of worse general cognitive resources. As language is the dominant medium for transmission and processing of information in humans (Kemmerer, 2014), it is likely that a necessary compensation for impaired speech perception is highly related to cognitive decline (Lin et al., 2013).

A similar theory—the information-degradation-theory—proposes that in elderly people, the de- creased sensory input and especially degraded speech perception is compensated by the recruitment of further cognitive resources. Thus, these resources cannot be used for higher cognitive tasks finally resulting in cognitive decline again, which might be reversible (Wayne and Johnsrude, 2015).

The central role of speech perception in cognitive decline is emphasized by the fact that worsened speech perception causes a cascade of secondary effects such as a loss of communicative skills, which consequently causes social isolation and finally depression (Amieva et al., 2015; Fortunato et al., 2016). In summary, it can be stated that no matter which hypothesis may explain the connection of hearing loss and cognitive decline best, speech perception might play a crucial role.

## Tinnitus, hearing loss, and stochastic resonance

It is a broad consensus that not only cognitive decline correlates with impaired hearing, but also tinnitus is mutually induced by or at least related to hearing loss (Konig et al., 2006; Savastano, 2008; Schaette and Kempter, 2012).

However, cochlear damage related to tinnitus does not necessarily lead to increased pure tone thresholds, but can be detected by electrophysiological measurements such as brainstem evoked response audiometry or speech in noise comprehension tests (Schaette and McAlpine, 2011; Paul et al., 2017; Bakay et al., 2018; Tziridis et al., 2021).

Note that, there exist many different models, which try to explain tinnitus development, including models based on e.g., decreased lateral inhibition (Gerken, 1996; Eggermont, 2003), thalamic gating (Rauschecker et al., 2010) or top-down models based on the Bayesian brain hypothesis (Sedley et al., 2016). However, here we want to focus on the two most advanced bottom-up models, which include already a solid mathematical and computational background: the central gain hypothesis (Norena, 2011; Auerbach et al., 2014) and the central noise hypothesis (Zeng, 2013, 2020).

According to the central gain model, tinnitus is caused by an increased sensitivity along the auditory pathway, in most cases as a result of homeostatic plasticity (Roberts, 2018), which increases the input sensitivity of neurons *via* several mechanisms. Unfortunately, this model only states that auditory input is somehow amplified, however, it does not explain, how the neural correlate of a persistent auditory phantom percept looks like.

A more recent model is the so called central noise model developed by Zeng (2013, 2020). In contrast to the central gain model, where the input signal is further amplified, the central noise model suggests that internally generated neural noise is added to the signal to increase the mean firing rates of neurons along the auditory pathway. However, it was not completely clear why internally generated neural noise is advantageous for auditory processing.

Starting in 2016, in a number of papers, we proposed an advancement of the central noise hypothesis (Krauss et al., 2016, 2017, 2018, 2019; Schilling et al., 2021a) and its consequences (Krauss and Tziridis, 2021; Schilling et al., 2022a). In particular, we proposed stochastic resonance to be the critical mechanism behind the central noise hypothesis. Thus, the auditory system mutually accounts for decreased cochlear input by the addition of neural noise, which stochastically lifts auditory signals above the detection threshold. Therefore, the addition of neural noise does counter- intuitively not mask the auditory input, but on the contrary improves the detection threshold (for details of the model see Krauss et al., 2016; Schilling et al., 2022b). Our model further suggests a feedback-loop in the cochlear nucleus, which continuously adjusts the amount of added noise by evaluating the information content of the neural signal. We could demonstrate that the signal's auto-correlation, may serve as a good estimate to quantify the amount of information (Krauss et al., 2017). Furthermore, the internally generated neural noise probably originates in the somato-sensory system, which is known to be connected to the dorsal cochlear nucleus (DCN) (Young et al., 1995; Shore and Zhou, 2006; Dehmel et al., 2012). Indeed cross-modal stochastic resonance is a universal principle to enhance sensory processing (Krauss et al., 2018) and can be found in different sensory modalities (cf. Manjarrez et al., 2007; Lugo et al., 2008; Ai et al., 2009; M'endez-Balbuena et al., 2015; Yashima et al., 2021). The stochastic resonance model predicts that the hearing ability is partly restored by the addition of the neural noise. Therefore, a slightly better hearing ability of tinnitus patients compared to an age-matched non-tinnitus group was a crucial prediction of our model, which we could actually confirm by analyzing patient data (Krauss et al., 2016; Gollnast et al., 2017), and with a newly developed animal paradigm to simulate transient hearing loss (Krauss and Tziridis, 2021). The improvement is around 5 dB pure-tone threshold decrease, which is not a huge benefit for every-day life. These insights eventually led us to develop a completely new treatment strategy for tinnitus: spectrally matched near-threshold noise significantly attenuated subjective tinnitus loudness in two pilot studies (Schilling et al., 2021b; Tziridis et al., 2022).

As described above, pure-tone audiometry is only one side of the whole truth. Tinnitus is always related to a measurable or a hidden hearing loss, which is more difficult to detect. A potential way to detect a hidden hearing losses is to check for the efficiency of the auditory system to process complex spectral temporal cues such as speech. Thus, hidden hearing loss is often diagnosed using speech or speech in noise comprehension tasks (Barbee et al., 2018; Monaghan et al., 2020). The stochastic resonance model suggests that the neural noise improves hearing and thus also leads to a better speech in noise comprehension. However, Oosterloo et al. (2020) found a decreased performance in a speech in noise detection task for people with tinnitus compared to a control-group without tinnitus. Also other studies point into this direction (Huang et al., 2007; Moon et al., 2015).

At a first glance this finding contradicts the central noise or stochastic resonance model, respectively. However, the mentioned studies report only a small effect size, which is only present at very high hearing losses above 25 dB. For lower hearing losses, the tinnitus group performs equally well or even slightly better than the non-tinnitus group.

Furthermore, many novel studies argue that in older studies the effect of hearing loss and tinnitus itself were not sufficiently decomposed. For example, Oosterloo et al. (2020) state “studies thus far have not been able to disentangle tinnitus, hearing loss, and speech in noise intelligibility”. Finally, Hamza and Zeng (2021) criticize that “most studies did not control for potential interactive factors such as age, sex, race, hearing loss, education, anxiety, depression, and physical wellbeing”.

To further investigate the effect of intrinsic neural noise on speech perception in an impaired auditory system, we have chosen a cognitive computational neuroscience (CCN) approach (Krauss and Schilling, 2020; Schilling et al., 2022b). Thus, we implemented a hybrid neural network, where the cochlea was modeled as parallel bandpass filters and the DCN as a layer of Leaky-Integrate-and-Fire (LIF) neurons. Higher brain structures were modeled as deep convolutional neural network (Schilling et al., 2022a). This hybrid neural network was trained on word recognition using a custom-made speech data set consisting of the 207 most common German words spoken by 10 different speakers, and the free spoken digit data set (FSDD) (Jackson et al., 2018). The combination of biologically inspired neuron models and brain inspired architectures with common deep learning architectures (Marblestone et al., 2016; Schutter, 2018; Richards et al., 2019; Tanaka et al., 2019; Krauss and Maier, 2020; Saxe et al., 2021; Yang et al., 2021; Maier et al., 2022; Schilling et al., 2022a), provides the possibility to use established techniques from artificial intelligence research (Krauss et al., 2012, 2021) on the one hand, and to analyze effects of impairments *in silico* (Gerum and Schilling, 2021) on the other hand. Thus, we showed that a simulated hearing loss, trivially leads to a decreased word recognition accuracy. However, this worsened accuracy caused by the simulated hearing loss could be re-improved by a factor of more than two by the addition of neural noise in the DCN (Schilling et al., 2022a). This study provides evidence that SR indeed can help to re-improve speech perception. Furthermore, it becomes obvious that the benefit of SR is far higher than a 5 dB decrease of pure-tone hearing thresholds. Thus, this simulation provides an answer to the question, why the implementation of the SR effect became evolutionary advantageous, although the secondary effect namely tinnitus leads to a significant drop in life quality and is a remarkable psychic burden.

## Discussion: Tinnitus, speech perception, and cognitive decline

There are three connections between tinnitus and speech perception—two causal and one correlational.

First, it is well known that (hidden) hearing loss induces tinnitus, and that hearing loss is also highly correlated with cognitive decline (Schaette and McAlpine, 2011). Furthermore, (hidden) hearing loss definitely impairs speech perception compared to normal hearing subjects. Therefore, tinnitus is correlated with impaired speech perception compared to subjects without hearing loss. However, there is not necessarily a causal relationship between tinnitus and impaired speech perception.

Second, we already showed that tinnitus improves hearing ability after hearing loss through the addition of neural noise (stochastic resonance) (Krauss et al., 2016). We also showed in a computational model that SR is also suited to enhance speech perception (Schilling et al., 2022a) These finding point to a positive effect of tinnitus on speech perception compared to patients suffering from hearing loss alone, but not compared to normal hearing subjects.

Third, tinnitus is also known to cause distress, which in turn causes concentration issues and attentional deficits, and hence might lead to a decrease of cognitive capacity and also speech perception (Ivansic and Guntinas-Lichius, 2017). Thus, tinnitus might also have a negative (side) effect on speech perception mediated by distress. However, these effects depend on the severity of the tinnitus percept and the subjectively perceived burden (Ivansic and Guntinas-Lichius, 2017).

These counteracting phenomena could explain the variety of findings, and tinnitus heterogeneiety. We conclude that internally generated neural noise can improve hearing ability and speech perception by means of SR. However, tinnitus leads to a significant psychic burden. Thus, people often report a decrease in the ability to focus on certain tasks (Hallam et al., 2004). Therefore, on the one hand psychic burden can favor cognitive decline, but on the other hand the improved speech perception abilities *via* SR can help to decrease cognitive decline *via* different pathways. Indeed, there exist some studies reporting a correlation of tinnitus and cognitive decline by means of mental concentration, executive control of attention, lower processing speed, or an impaired short term memory (Hallam et al., 2004; Savastano, 2008; Tegg-Quinn et al., 2016; Chu et al., 2020; Clarke et al., 2020). However, recently Hamza and Zeng (2021) reported a decreased cognitive decline in tinnitus patients compared to an age-matched control group. Thus, the authors speculate that in some earlier studies the effect of tinnitus on cognitive decline is overestimated and mixed up with effects of hearing loss. We argue that all of these findings can be brought together with no further contradictions. On the one hand tinnitus indeed causes cognitive decline *via* secondary effects such as psychic burden, depression and difficulties in concentration. On the other hand, tinnitus has also a benefit on cognitive performance (Hamza and Zeng, 2021), as tinnitus improves speech perception and decreases the hearing thresholds and therefore symptoms of worsened speech perception such as social isolation are reduced.

## Author contributions

All authors listed have made a substantial, direct, and intellectual contribution to the work and approved it for publication.
